# Neurogenic Paradoxical Vocal Fold Motion: A Systematic Review of Central and Pheripheral Nervous System Disorders

**DOI:** 10.1016/j.bjorl.2026.101894

**Published:** 2026-09-09

**Authors:** Bigyan Raj Gyawali, Bibek Shrestha, Shreeram Paudel, Rashmi Gupta

**Affiliations:** a Tribhuvan University Teaching Hospital, Department of Ear, Nose, Throat, Head and Neck Surgery, Kathmandu, Nepal; b Tribhuvan University, Institute of Medicine, Maharajgunj Medical Campus, Kathmandu, Nepal

**Keywords:** Vocal cord dysfunction, Paradoxical vocal fold motion, Neurogenic laryngeal dysfunction, Neurological disorders, Stridor

## Abstract

**Objective(s):**

To systematically synthesize published evidence on paradoxical vocal fold motion associated with confirmed central or peripheral neurological disorders, with emphasis on neuroanatomic correlations, clinical presentation, management strategies, and treatment outcomes.

**Methods:**

A systematic review was conducted following PRISMA 2020 guidelines and registered in PROSPERO. Six databases (PubMed/MEDLINE, Embase, Scopus, Cochrane Library, ScienceDirect, and Google Scholar) were searched from inception to December 2025. Studies reporting PVFM associated with a confirmed neurological disorder in patients of any age were included. Eligible designs comprised case reports, case series, and observational studies. Data extraction was performed using predefined criteria, and risk of bias was assessed using Joanna Briggs Institute (JBI) critical appraisal tools and the ROBINS-I framework.

**Results:**

Eleven studies were included, enrolling 102 patients, of whom 42 (19 pediatric, 23 adult) had paradoxical vocal fold motion in association with a confirmed neurological disorder. Neurological etiologies encompassed brainstem malformations, epilepsy, cerebral palsy, neuromuscular junction disorders, basal ganglia diseases, neurodegenerative disorders, and ion-channelopathies. The review design precluded estimation of the prevalence of neurological causes among all PVFM cases; within individual cohorts, neurological disease was identified in 54% of infants with PVFM, and paradoxical vocal fold motion was documented in 33% of patients with multiple system atrophy. Six of the nine studies with classifiable etiologies involved reversible or treatable conditions and three involved progressive neurodegenerative disease, although most individual patients fell into the latter group because the neurodegenerative cohorts were larger. Treatment directed at the underlying neurological disorder resulted in substantial improvement or resolution in most reversible cases, whereas symptoms often persisted in progressive diseases.

**Conclusion:**

PVFM is frequently associated with organic neurological disorders and should be recognized as a manifestation of disrupted neural control rather than solely a functional condition. Early neurological evaluation and disease-specific management may improve outcomes, particularly in atypical or refractory presentations.

## Highlights

• PVFM often linked to organic neurological disorders, not purely functional.
• Causes include brainstem malformations, epilepsy, and NMJ disorders.
• Neurological disease seen in up to 54% of infants with PVFM.
• ∼60%–70% cases reversible with improvement after disease-specific therapy.
• Findings support a neurologically oriented diagnostic approach for PVFM.

## Introduction

Vocal cord dysfunction, also known as paradoxical vocal fold motion, refers to inappropriate vocal fold adduction during inspiration. Patients often present with throat discomfort, dysphagia, inspiratory stridor and acute respiratory distress which can mimic as asthma or other obstructive lung disease.^1^ Due to presence of overlapping and transient symptoms, PVCD often gets misdiagnosed and mistreated leading to repeated hospitalizations and inappropriate use of corticosteroid therapy or bronchodilators.^2^ Most often, PVCD is contextualized as psychogenic or functional disorder as existing literature frames PVCD as paradigms of anxiety, stress or maladaptive laryngeal behavior following which, the diagnostic pathway of PVCD has been exclusion of structural pathology rather than identifying the existing and underlying mechanisms.^3^ Functional PVCD is a well-recognized entity; however, this dominant perspective may overlook underlying organic causes, particularly in patients with atypical presentations, refractory symptoms, or associated neurological abnormalities.^4^^,^^5^

A growing literature has recorded PVFM to have a relationship with various neurological conditions.^5^^,^^6^ Etiologies reported include compression of the brainstem, Arnold Chiari malformation and hydrocephalus, neurodegenerative diseases, multiple system atrophy, epileptic and supranuclear motor disorders, basal ganglia disorder and peripheral neuromuscular junction or ion-channel diseases.^7^^,^^8^ These links are especially evident in the pediatric and neurologically complicated groups, with a significant number of PVFM cases having an underlying discernible central or peripheral nervous system pathology. Notably, several studies have also shown that PVFM gets improved or resolved when the underlying neurological disorder is treated specifically, which correlates with the causality between the two instead of coincidental cooccurrence.^9^^,^^10^ Neurogenic PVFM should be recognized with clinical implications. In this regard, PVFM can be used as a neurological manifestation of dysfunctional laryngeal motor control, not as a diagnosis of exclusion.^11^ In patients with neurological disease, PVFM may reflect the severity, progression, or reversibility of the underlying disorder, depending on the neuroanatomic site and mechanism involved. In addition, therapeutic reactions to neuromuscular-specific therapy, neurosurgical decompression, and seizure control, and cerebral fluid diversion all support the pathophysiologic connection between neurological pathology and paradoxical vocal fold movement.^11^^,^^12^

Regardless of these observations, the neurological aspect of PVFM is not synthesized well enough. The different literature has covered small case studies and small observational cohorts, however there is no systematic review that has directly investigated PVFM in reference to organic neurological etiologies. Recognition of a neurogenic basis for PVFM also has important therapeutic implications. In contrast to functional or psychogenic PVFM, which is primarily managed with behavioral therapy and respiratory retraining, neurogenic PVFM often reflects an underlying structural, neurodegenerative, or neuromuscular disorder affecting the laryngeal motor control network.^13^ In several reported cases, targeted treatment of the underlying neurological condition such as neurosurgical decompression, cerebrospinal fluid diversion, antiepileptic therapy, or disease specific neuromuscular treatment has resulted in substantial improvement or complete resolution of paradoxical vocal fold motion.^14^, ^15^, ^16^ These observations suggest that PVFM may serve not only as a respiratory manifestation of neurological disease, but also as a potentially reversible clinical sign when the underlying pathology is identified early. Failure to recognize neurogenic PVFM may therefore delay appropriate neurological evaluation, expose patients to unnecessary respiratory treatments, and miss opportunities for disease specific intervention.^17^

Neuroanatomic correlations, clinical phenotypes, and treatment outcomes have not been integrated into a unified framework, limiting mechanistic understanding and clinical translation. The present systematic review aims to address this gap by systematically evaluating neurological disorders associated with PVFM. By focusing on organic central and peripheral nervous system pathology, this review seeks to delineate neuroanatomic correlations, characterize clinical presentations across age groups, and assess outcomes following neurological and airway-directed interventions. Through this approach, we aim to reframe PVFM within a neurologically informed diagnostic and therapeutic paradigm.

## Methods

**Fig. 1:**
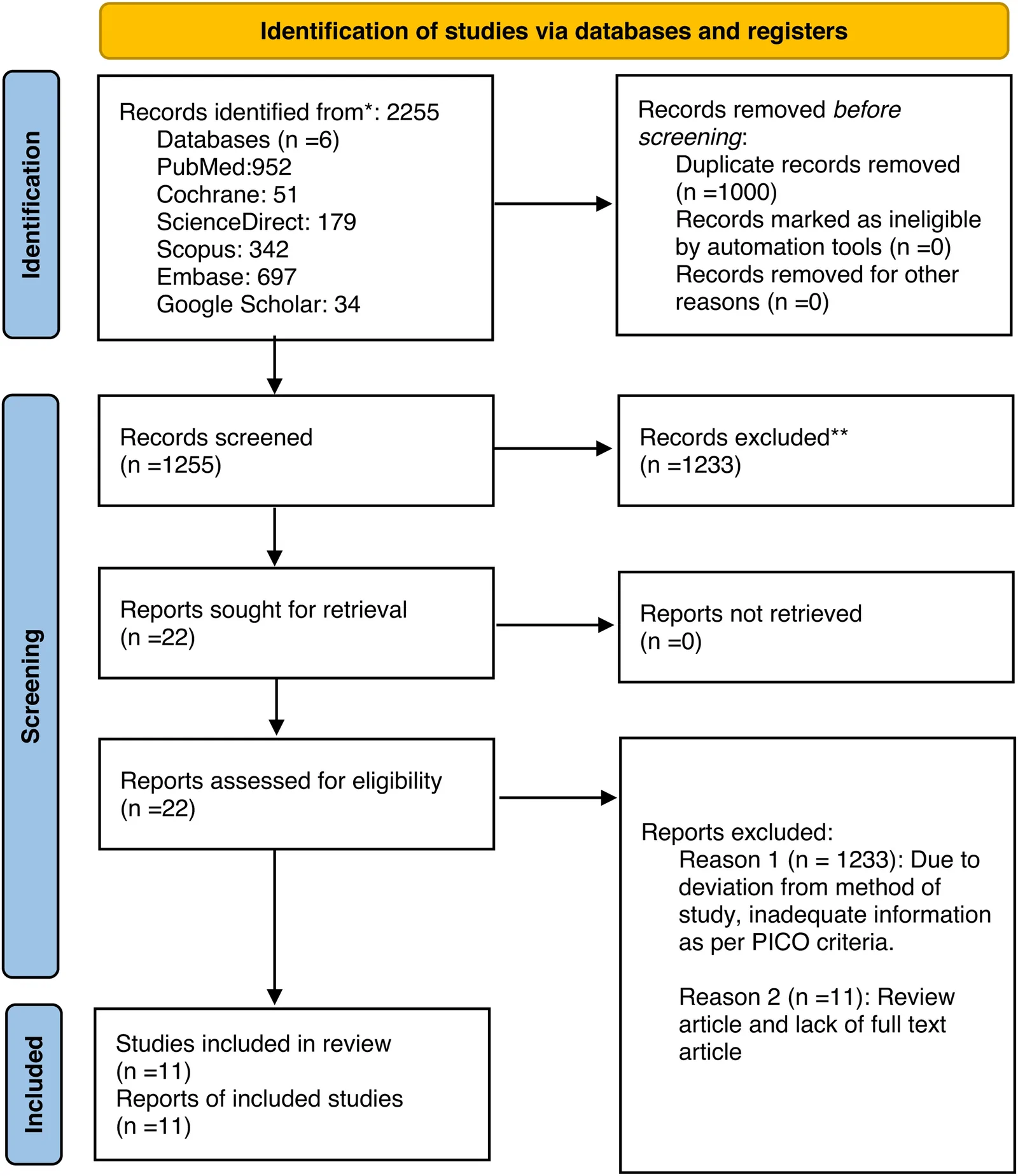
PRISMA flow diagram of study selection process. Source: Page MJ, et al. BMJ 2021;372:n71. doi: 10.1136/bmj.n71. This work is licensed under CC BY 4.0. To view a copy of this license, visit https://creativecommons.org/licenses/by/4.0/.

This systematic review was conducted in accordance with the Preferred Reporting Items for Systematic Reviews and Meta-Analyses (PRISMA) 2020 guidelines.^18^ A predefined protocol guided the methodology, including literature search strategy, eligibility criteria, study selection, data extraction, and qualitative synthesis was registered in PROSPERO. The methodological quality of this systematic review was assessed using the AMSTAR-2 (A MeaSurement Tool to Assess systematic Reviews 2) checklist, which is designed to evaluate the rigor and credibility of systematic reviews that include randomized and non-randomized studies of interventions.^19^ The study aimed to systematically evaluate the neurological disorders associated with PVFM and to describe their clinical characteristics, neuroanatomic correlations, management strategies, and outcomes. The study selection process is summarized in the PRISMA flow diagram (Fig. 1).

A comprehensive literature search was performed using both controlled vocabularies, where available, and free-text terms related to PVFM and vocal cord dysfunction. Search terms included combinations of: ‘paradoxical vocal fold motion’, ‘paradoxical vocal fold movement’, ‘paradoxical vocal cord motion’, ‘vocal cord dysfunction’, ‘inducible laryngeal obstruction’, and neurological terms including ‘neurologic’, ‘neurological disorder’, ‘brainstem’, ‘Chiari malformation’, ‘epilepsy’, ‘multiple system atrophy’, ‘myasthenia gravis’, and ‘neuromuscular disease’. Database-specific syntax was adapted for PubMed/MEDLINE, Embase, Scopus, Cochrane Library, ScienceDirect, and Google Scholar. Studies were selected according to predefined PICO-based inclusion criteria. Eligible studies included original research reporting patients of any age diagnosed with PVFM or paradoxical vocal cord dysfunction in association with a confirmed neurological disorder. Acceptable study designs included case reports, case series, retrospective studies, and prospective observational studies. Only studies published in English with available full text were considered. Studies were excluded if PVFM was attributed solely to non-neurological causes (e.g., psychogenic, reflux-related, or purely functional etiologies), if the neurological diagnosis was unclear or unsupported, or if the publication was a review article, editorial, or animal study.

All records retrieved from the database search were imported into a reference management system, and duplicate records were removed prior to screening. Titles and abstracts were independently screened for relevance by three reviewers (B.S., S.P., and R.G.). Full-text articles were subsequently assessed for eligibility based on the predefined inclusion and exclusion criteria. Disagreements during the screening process were resolved through discussion and consensus, with arbitration by a fourth author when required. Inter-reviewer agreement at both the title/abstract and full-text screening stages was assessed using Cohen’s kappa statistic, and agreement levels were interpreted as slight (0.00–0.20), fair (0.21–0.40), moderate (0.41–0.60), substantial (0.61–0.80), and almost perfect (0.81–1.00).^20^ Of the 2,255 records initially identified, 1,000 duplicates were removed, leaving 1,255 records for screening. After title and abstract review, 1,233 records were excluded due to irrelevance or failure to meet eligibility criteria. Twenty-two full-text articles were assessed, of which 11 studies fulfilled all inclusion criteria and were included in the final qualitative synthesis. Data extraction was performed using a standardized data collection form. Extracted variables included: study characteristics (author, year, country, study design), patient demographics (age, sex), type of neurological disorder, presumed neuroanatomic level of involvement, clinical manifestations of PVFM, diagnostic methods, treatment strategies, and reported outcomes. When multiple patients were reported within a single study, data were extracted at both the study and individual patient levels when possible.

Given the predominance of descriptive and non-randomized study designs, risk of bias was assessed using tools appropriate to study type rather than a single quantitative instrument. Case reports and case series were appraised using the Joanna Briggs Institute (JBI) Critical Appraisal Checklists, which evaluate methodological quality across domains including clarity of patient demographics, validity of diagnostic methods, completeness of clinical history, adequacy of intervention description, and transparency of outcome reporting.^21^ For non-randomized observational studies (retrospective and prospective cohort designs), risk of bias was assessed using the Risk Of Bias In Non-randomized Studies of Interventions (ROBINS-I) tool.^22^ Each study was judged qualitatively as having low, moderate, serious, or critical risk of bias based on these criteria. Because the evidence included largely comprised rare disease reports and small observational cohorts, risk-of-bias assessments were used to inform the interpretation and strength of conclusions, rather than to exclude studies from the synthesis.

Due to substantial clinical heterogeneity in study design, patient populations, neurological etiologies, and outcome measures, a meta-analysis was not feasible. Therefore, a qualitative narrative synthesis was performed. Findings were grouped and analyzed according to neurological etiology, neuroanatomic involvement, clinical presentation, and response to treatment. Patterns of association and consistency of outcomes were emphasized to support evidence-based interpretation.

## Results

A total of 11 studies were included in this systematic review, comprising 102 patients with PVFM associated with an underlying neurological disorder.^14^, ^15^, ^16^^,^^23^, ^24^, ^25^, ^26^, ^27^, ^28^, ^29^, ^30^ The evidence base consisted predominantly of case reports and small case series, alongside three prospective or retrospective observational cohorts, reflecting the rarity and clinical heterogeneity of neurogenic PVFM. The included studies spanned pediatric and adult populations and originated from North America and Europe (Table 1).

**Table 1:** Study characteristics of included studies.

Study	Year	Country	Design	Sample size	Population	Age	Sex	Neurologic diagnosis
Gross JH et al.	2017	USA	Case report	1	Pediatric patient presenting with paradoxical vocal fold motion and apnea	9 years	Male	Benign Rolandic Epilepsy (focal onset epilepsy with left-central epileptiform discharges on EEG)
Pascal et al.	2025	USA	Retrospective Study Design	24	Infants (0–18 months) with paradoxical vocal fold motion	Mean 3.4 months (range 0–15)	63% male	54% had neurologic disorders: cerebral palsy, epilepsy, posterior fossa ependymoma, global developmental delay, myelomeningocele, hydrocephalus, neonatal abstinence syndrome
Cleveland CN et al.	2020	USA	Case Report	1	Infant with paradoxical vocal cord motion progressing to bilateral vocal cord paresis	Neonate → 15 months follow-up	Female	Chiari II malformation with hydrocephalus and VP shunt; brainstem compression affecting vagal motor nuclei
Portier F et al.	2001	France	Retrospective case series	6	Infants with Chiari I or II malformations and laryngeal respiratory obstruction	Neonates to ∼45 days at presentation; mean follow-up 30 months	4 male, 2 female	Laryngoscopy under anesthesia; PVCM defined as inspiratory vocal-cord adduction Chiari malformation with brainstem dysfunction (5 Chiari II with myelomeningocele, 1 Chiari I)
Todisco T et al.	2000	Italy	Case Report	1	Adult with acute inspiratory stridor due to paradoxical vocal cord dysfunction	26 years	Male	Myasthenia gravis (ACh-receptor antibody positive; EMG confirmed)
Gandor F et al.	2020	Germany	Prospective observational cohort	57 MSA (57 PD controls)	Adults with multiple system atrophy compared with Parkinson’s disease	MSA median 64y (IQR 59–71)	35 Female and 22 Male	Multiple system atrophy (parkinsonian or cerebellar phenotypes)
Rafizadeh et al.	2013	USA	Case Report	1	Adult with intermittent stridor and paradoxical vocal fold motion	32 years	Female	Pantothenate kinase-associated neurodegeneration (PKAN) due to PANK2 mutation
Warnecke T. et al.	2019	Germany	Prospective pilot study	8 MSA patients	Adult with multiple system atrophy	Mean 59.9 ± 6.9 years	4 female, 4 male	Multiple system atrophy (7 MSA-P, 1 MSA-C)
Bally K., Railwah D.	2017	USA	Case Report	1	Adult patient presenting with asthma-like symptoms and paradoxical vocal cord dysfunction	27 years	Male	Pituitary macroadenoma (prolactinoma) with skull-base erosion and CSF leak
Purkey MR & Valika T	2019	USA	Case Report	1	Neonate with recurrent stridor, apnea and PVFM	1 day old (term neonate, 40 weeks gestation)	Female	Paramyotonia congenita due to SCN4A mutation (neuromuscular sodium-channel disorder)
Cheng YS et al.	2013	UK	Case Report	1	Child with chronic stridor and episodic airway obstruction	3.5 years	Male	Spastic quadriplegic cerebral palsy (with MRI-documented white-matter injury)

Neurological causes of PVFM encompassed a broad spectrum of central and peripheral nervous system disorders. In pediatric populations, PVFM was most frequently associated with structural brainstem pathology (Chiari I/II malformations with or without hydrocephalus), epileptic disorders, cerebral palsy, and congenital neuromuscular channelopathies. In adults, PVFM occurred in the context of neurodegenerative disease, particularly Multiple System Atrophy (MSA), autoimmune neuromuscular junction disorders, basal ganglia disorders, and central mass lesions.

Because this review specifically included studies in which PVFM was associated with a confirmed neurological disorder, the included evidence does not permit estimation of the overall prevalence of neurological etiologies among all PVFM or vocal cord dysfunction cases. However, several included cohort studies reported neurological comorbidity within selected PVFM populations, including infant and pediatric cohorts, suggesting that neurological evaluation may be particularly relevant in early-onset, atypical, or refractory presentations.^15^ The implicated neuroanatomic substrates clustered around laryngeal motor control pathways, including the nucleus ambiguus, vagal efferent fibers, brainstem respiratory centers, basal ganglia, and supranuclear cortical networks. Disorders affecting the brainstem either structurally (Chiari malformation, hydrocephalus) or functionally (MSA) ‒ demonstrated the most consistent association with inspiratory vocal fold adduction.

Clinically, inspiratory stridor was the predominant presenting symptom across age groups, frequently accompanied by dyspnea, feeding-related choking, apneic or cyanotic episodes, and asthma-like attacks. Pediatric patients are more commonly presented with feeding difficulties and apnea, whereas adults frequently exhibited exercise- or anxiety-induced stridor. Laryngoscopic or endoscopic evaluation consistently demonstrated paradoxical inspiratory adduction of the vocal folds with preserved or near-normal expiratory abduction, confirming the diagnosis of PVFM.

Therapeutic strategies were directed primarily at the underlying neurologic disorder, supplemented by supportive airway interventions. In conditions with reversible or treatable neurologic pathologies such as epilepsy, hydrocephalus, Chiari malformation, myasthenia gravis, and ion-channel disorders targeted medical or surgical treatment resulted in substantial improvement or complete resolution of PVFM. Surgical decompression in Chiari malformation and cerebrospinal fluid diversion for hydrocephalus was particularly effective, with sustained normalization of airway function in most cases.

Conversely, in progressive neurodegenerative conditions such as MSA, PVFM tended to persist despite symptomatic improvement. Continuous Positive Airway Pressure (CPAP) provided meaningful relief of stridor and dyspnea, but did not eliminate paradoxical vocal fold motion, reflecting irreversible neurodegenerative involvement of laryngeal control pathways. Botulinum toxin injection into intrinsic laryngeal muscles was effective in selected pediatric and spastic conditions, allowing avoidance of tracheostomy. Overall, outcomes were most favorable when early recognition of neurogenic PVFM led to timely neurologic intervention, underscoring the importance of considering central and peripheral neurologic etiologies in patients with refractory or atypical PVFM presentations (Table 2, Table 3).

**Table 2:** Neurological etiology and clinical manifestations.

Study	Neurologic disorder	Neuro-anatomic level	Neuro-PVFM burden (Incidence form)	PVFM manifestations
Gross JH et al., 2017	Benign Rolandic epilepsy (focal onset epilepsy)	Rolandic cortex (left central region); insular-oropharyngolaryngeal cortical network	1/1	Episodic inspiratory stridor, aphonia, dyspnea, throat tightness, laryngeal spasm-like apnea
Pascal ES et al., 2025	Mixed neurologic disorders: cerebral palsy, epilepsy, posterior fossa ependymoma, global developmental delay, myelomeningocele, hydrocephalus, neonatal abstinence syndrome	Central nervous system (cortex, brainstem, posterior fossa; vagal-laryngeal control pathways)	13 of 24 PVFM infants had neurologic disease: 54% neurologic-associated PVFM	Chronic stridor (96%), feeding-triggered stridor, coughing with feeds, dysphagia, laryngeal penetration, inducible inspiratory vocal-fold adduction
Cleveland CN et al., 2020	The infant had hydrocephalus, myelomeningocele and Chiari II malformation requiring VP shunt	MRI showed cerebellar ectopia (Chiari II) with brainstem compression, affecting vagal motor nuclei (nucleus ambiguus) which control vocal fold movement	1 of 1 infant with Chiari II had PVFM → 100% neurologic-PVFM in this report	Inspiratory stridor, feeding-related choking, paradoxical vocal fold adduction on laryngoscopy, episodic cyanosis, obstructive sleep apnea; later progression to bilateral vocal cord paresis
Portier F et al., 2001	Chiari malformation with brainstem dysfunction (5 Chiari II with myelomeningocele ± hydrocephalus; 1 Chiari I)	Brainstem (medulla & vagal nuclei) compressed at foramen magnum	2 of 6 children (33%) had PVCM among those with Chiari-related laryngeal obstruction	Inspiratory stridor, episodic airway obstruction, paradoxical inspiratory vocal-cord adduction on endoscopy, feeding-related choking, apnea
Todisco T et al., 2000	Myasthenia Gravis	Peripheral neuromuscular junction of intrinsic laryngeal muscles (ACh-receptor-mediated fatigability of posterior cricoarytenoid abductors)	1 of 1 MG patient (100%) had PVFM in this report	Acute inspiratory stridor, episodic airway obstruction, paradoxical inspiratory vocal-cord adduction with normal expiratory abduction, asthma-like attacks
Gandor F et al., 2020	Multiple system atrophy (MSA) – parkinsonian and cerebellar phenotypes	Brainstem & basal ganglia–laryngeal motor network (nucleus ambiguus, striatonigral and olivopontocerebellar systems)	PVFM present in 19/57 MSA patients = 33.3%	Paradoxical vocal-fold adduction during inspiration and paradoxical abduction during expiration on FEES; often accompanied by inspiratory stridor, dyspnea, dysphonia
Rafizadeh S & Long JL, 2013	Pantothenate kinase-associated neurodegeneration (PKAN) due to PANK2 mutation	Basal ganglia (globus pallidus) with extrapyramidal & autonomic laryngeal control dysfunction	1 of 1 PKAN patient (100%) had PVFM	Paradoxical vocal fold adduction during inspiration. Normal abduction during expiration and phonation. Clinically: Intermittent stridor; Worse with activity or anxiety; Absent during sleep
Warnecke T. et al., 2019	Multiple system atrophy (MSA) – parkinsonian (MSA-P) and cerebellar (MSA-C) subtypes	Brainstem (nucleus ambiguus), basal ganglia & cerebellar–laryngeal motor network	PVFM in 1 of 8 MSA patients = 12.5%	Paradoxical vocal-fold adduction during inspiration on FEES; often accompanied by stridor, dysphonia, and irregular arytenoid cartilage movement
Bally K & Railwah D, 2017	Pituitary macroadenoma (prolactinoma) with skull-base erosion and CSF leak	Central skull base & hypothalamic-pituitary region with secondary brainstem/autonomic laryngeal control disruption	1 of 1 patient (100%) had PVFM	Intermittent inspiratory stridor, aphonia, air hunger, paradoxical inspiratory vocal-cord adduction on laryngoscopy
Purkey MR & Valika T., 2019	Paramyotonia congenita due to SCN4A sodium-channel mutation	Peripheral neuromuscular junction & intrinsic laryngeal muscles (laryngeal myotonia)	1 of 1 patient (100%) had PVFM	Episodic stridor progressing to apnea and bradycardia; paradoxical inspiratory vocal-fold adduction with normal motion between episodes
Cheng YS et al., 2013	Spastic quadriplegic cerebral palsy with diffuse white-matter injury	Supranuclear & brainstem laryngeal motor control pathways	1 of 1 patient (100%) had PVFM	Recurrent inspiratory stridor, episodic airway obstruction, paradoxical inspiratory vocal-fold adduction on microlaryngoscopy

**Table 3:** Treatment and outcomes of neurogenic PVFM.

Study	Neurologic intervention	Laryngeal response	Outcome
Gross JH et al., 2017	Antiepileptic therapy: levetiracetam initiated, followed by addition and titration of oxcarbazepine	Marked reduction of PVFM-like laryngeal events; apnea and stridor shortened from 30–60 s to ∼10 s, then resolved	Complete seizure and PVFM-like symptom control; seizure-free for > 5 weeks
Pascal ES et al., 2025	Treatment of underlying neurologic disease (antiepileptics, NAS medical therapy, neurosurgical management of hydrocephalus/tumor) plus supportive airway and feeding management	Reduction in PVFM severity as neurologic condition stabilized; neurologic patients showed slower and less complete resolution of paradoxical vocal fold motion	Neurologic PVFM associated with prolonged symptoms (mean 15.5-months) and high feeding-tube dependence; some NAS and medically treated patients regained oral feeding
Cleveland CN et al., 2020	VP shunt placement at birth and multiple shunt revisions for hydrocephalus and Chiari II malformation	PVFM and later bilateral vocal cord paresis worsened with shunt malfunction	No residual stridor
			Near-normal polysomnography (AHI 1.4)
	Antiepileptic treatment (oxcarbazepine) for seizure-like episodes	After shunt revision, bronchoscopy showed return of vocal cord motion and resolution of stridor	Tracheostomy avoided
Portier F et al., 2001	Posterior fossa decompression (brainstem decompression) after stabilization of hydrocephalus ± shunting; temporary tracheostomy or feeding tube as supportive care	Resolution of PVCM and laryngeal obstruction after decompression (within days to months); recurrence with raised intracranial pressure	All 5 decompressed children had full functional recovery of airway, swallowing, and voice
			The only child not decompressed had persistent stridor and feeding difficulty for > 9 years
Todisco T et al., 2000	nCPAP (10 cm H_2_O) relieved acute laryngeal obstruction	Stridor ceased after nCPAP	No recurrence of PVFM
	Pyridostigmine + prednisone started after MG diagnosis	Flow-volume loop and MEF50/MIF50 ratio normalized (9.9 → 1.36)	No need for intubation or tracheostomy
			3 years later, patient well and off therapy
	Thymectomy performed, improving disease course	Laryngoscopy showed resolution of paradoxical adduction	
Gandor F et al., 2020	Continuous positive airway pressure (CPAP) and neurologic supportive therapy for MSA (dopaminergic and autonomic management)	CPAP reduced inspiratory collapse and PVFM severity; FEES showed improved vocal fold patency during inspiration	Symptom relief (less stridor and dyspnea) but PVFM persisted because of progressive neurodegeneration
Rafizadeh S & Long JL, 2013	Expectant management (no immediate intervention); future options discussed: botulinum toxin or tracheostomy	PVFM persisted but remained mild and intermittent; no progression during observation	Stable disease: no functional respiratory compromise botulinum toxin or tracheostomy were considered but not required
Warnecke T. et al., 2019	Continuous positive airway pressure (CPAP) for nocturnal stridor; standard MSA neurologic management	Improved airway patency and reduced inspiratory PVFM during sleep; FEES abnormalities persisted	Patients continued to have progressive neurodegenerative disease, with airway symptoms controlled but not cured
Bally K & Railwah D, 2017	Bromocriptine therapy for prolactinoma; evaluation and management of CSF leak	Improvement of PVFM symptoms (reduction in dyspnea, choking, and aphonia) as tumor-related endocrine and pressure effects treated	Resolution of PVCD with medical therapy; avoidance of unnecessary asthma escalation
Purkey MR & Valika T., 2019	Carbamazepine (sodium-channel blocker) plus reflux control	Rapid resolution of PVFM and stridor; vocal folds remained mobile between episodes	Sustained improvement; minimal respiratory episodes at 1-year follow-up
Cheng YS et al., 2013	Periodic botulinum toxin type-A injections into thyroarytenoid and lateral cricoarytenoid muscles	Rapid resolution of inspiratory PVFM and stridor within 3 days; recurrence controlled by repeat injections every 3–4 months	At latest follow-up the child was free of airway obstruction with no aspiration and no tracheostomy

**Fig. 2:**
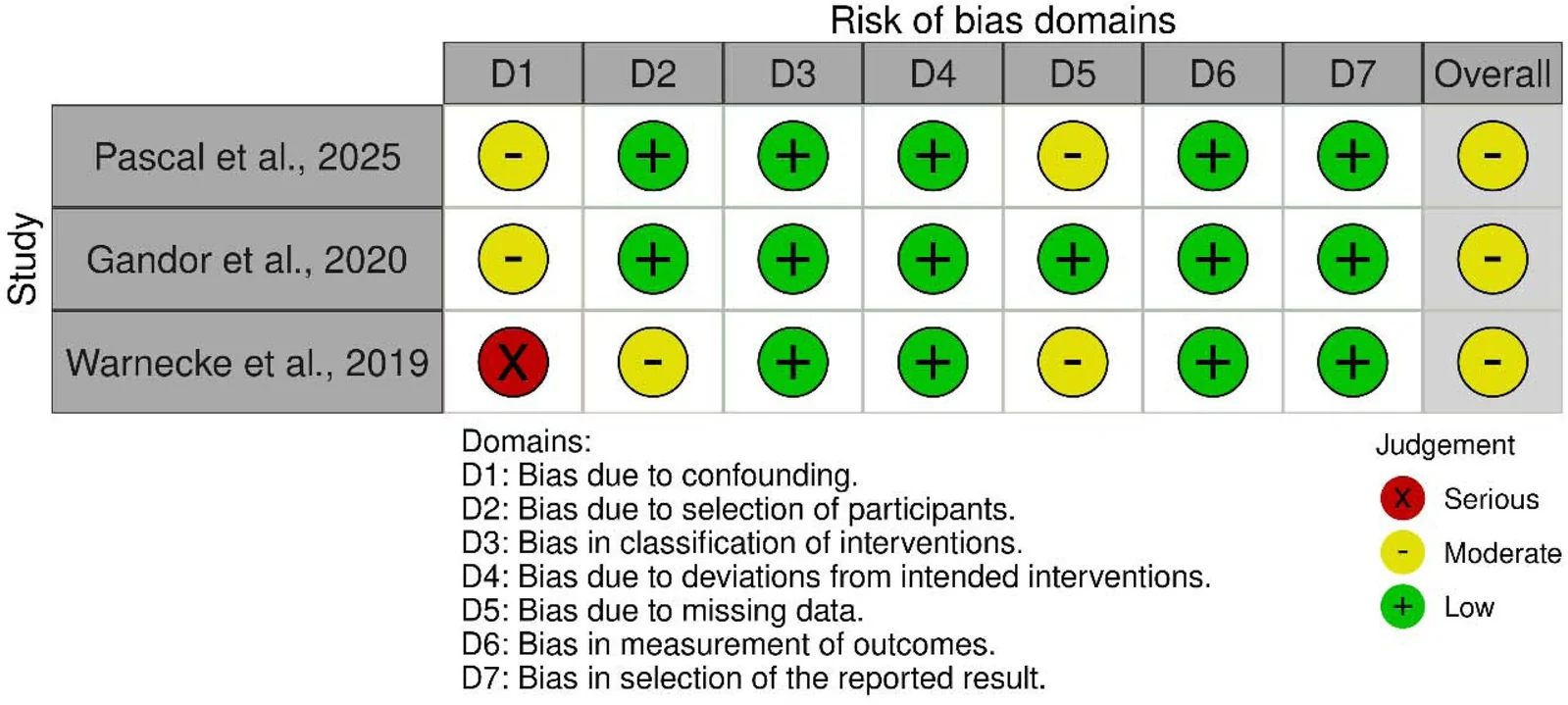
Risk of bias domains for included observational studies (ROBINS-I traffic-light plot).

**Fig. 3:**
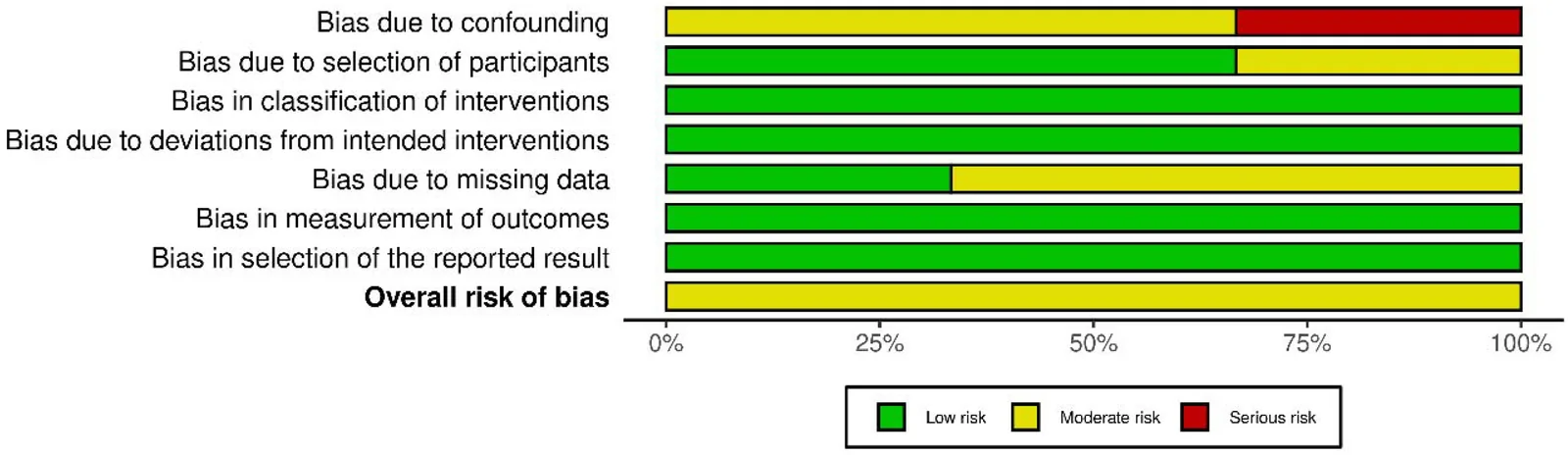
Summary of risk of bias across domains for observational studies.

Risk of bias was assessed according to study design using the JBI critical appraisal checklists for case reports and case series, and the ROBINS-I tool for observational cohort studies (Table 4, Table 5, Table 6). The traffic plot and summary plot are presented in Fig. 2 and Fig. 3. Among the seven included case reports, methodological quality was generally high. Five studies fulfilled seven or more JBI domains and were judged to have low risk of bias, while two studies were classified as moderate risk, primarily due to incomplete reporting of adverse events or limited detail regarding intervention outcomes. No case report was judged to have high risk of bias. The single included case series demonstrated an overall moderate risk of bias. Although diagnostic methods, patient characteristics, and outcomes were clearly reported, the study lacked explicit documentation of consecutive or complete case inclusion and did not perform formal statistical analysis, which reduced methodological robustness.

**Table 4:** JBI risk of Bias assessment for included case reports.

Study	Demographics clearly described	Patient history clearly described	Timeline clearly described	Diagnostic methods appropriate	Intervention clearly described	Post-intervention condition described	Adverse events described	Take-away lessons provided	Overall ROB
Gross et al., 2017	Yes	Yes	Yes	Yes	Yes	Yes	Yes	Yes	Low
Cleveland et al., 2020	Yes	Yes	Yes	Yes	Yes	Yes	Yes	Yes	Low
Todisco et al., 2000	Yes	Yes	Yes	Yes	Yes	Yes	No	Yes	Low
Rafizadeh et al., 2013	Yes	Yes	Yes	Yes	No	Yes	No	Yes	Moderate
Bally & Railwah, 2017	Yes	Yes	Yes	Yes	Yes	Yes	No	Yes	Low
Purkey & Valika, 2019	Yes	Yes	Yes	Yes	Yes	Yes	Yes	Yes	Low
Cheng et al., 2013	Yes	Yes	Yes	Yes	Yes	Yes	No	Yes	Low

**Table 5:** JBI risk of Bias assessment for included case series.

Study	Clear inclusion criteria	Standard condition measurement	Valid diagnostic methods	Consecutive inclusion	Complete inclusion	Clear demographics	Clear clinical information	Outcomes reported	Site demographics reported	Appropriate statistics	Overall ROB
Portier et al., 2001	Yes	Yes	Yes	Unclear	Unclear	Yes	Yes	Yes	Yes	No	Moderate

**Table 6:** ROBINS-I risk of Bias assessment for observational studies.

Study	Confounding	Selection of participants	Classification of exposure	Deviations from intended exposure	Missing data	Outcome measurement	Selective reporting	Overall ROB
Pascal et al., 2025	Moderate	Low	Low	Low	Moderate	Low	Low	Moderate
Gandor et al., 2020	Moderate	Low	Low	Low	Low	Low	Low	Moderate
Warnecke et al., 2019	Serious	Moderate	Low	Low	Moderate	Low	Low	Moderate

Among the three observational cohort studies, three were judged to have a moderate risk of bias according to the ROBINS-I tool. The principal sources of bias were confounding due to disease severity and comorbid neurologic conditions, small sample sizes, and incomplete follow-up in some cohorts. Outcome measurement and exposure classification were generally reliable across studies, as most used objective laryngoscopic or endoscopic confirmation of paradoxical vocal fold motion. Overall, the evidence included was dominated by case reports and small observational cohorts, resulting in predominantly low-to-moderate methodological quality, with a minority of studies demonstrating serious risk of bias. These assessments were considered during interpretation of findings but were not used as exclusion criteria, given the rarity and clinical heterogeneity of neurogenic PVFM.

## Discussion

The systematic review summarizes the available evidence of a causal relationship between PVFM and organic neurological disorders and proves that neurogenic mechanisms are a considerable and clinically significant subgroup of PVFM in both children and adults. This review (combining data of 11 studies including 102 patients) suggests a broad range of central and peripheral nervous system disorders that are related to PVFM, represents consistent neuroanatomic systems underlying aberrant motor control of the larynx, and emphasizes the prognostic and therapeutic significance of curing the underlying neurological pathology. All this evidence suggests reconsideration of existing diagnostic frameworks, in which PVFM is not viewed solely as a functional or psychogenic disorder, but also as a potential manifestation of disrupted neural control of the larynx.

One of the key findings of this review is the diversity of neurological disorders reported in association with PVFM, including structural brainstem disorders, epileptic disorders, neurodegenerative disease, neuromuscular junction disorders, basal ganglia disease, and ion-channel disorders. Importantly, this systematic review was not designed to determine the population prevalence of neurological causes among all patients with vocal cord dysfunction or PVFM. Rather, it aimed to synthesize the clinical and mechanistic evidence from studies in which a neurological disorder was confirmed. Pediatric PVFM most frequently followed the pathology of the brainstem or posterior fossa, epilepsy, cerebral palsy, and congenital neuromuscular disorders, whereas the pathology of the adult PVFM mainly involved neurodegenerative diseases including MSA, autoimmune neuromuscular junction disorders, and focal central lesions.^31^^,^^32^ This developmental effect may be due to age-related developmental susceptibility of laryngeal motor control networks during infancy and early childhood and mature degeneration or local disruption of established motor pathways in adults.^33^^,^^34^ Importantly, the high prevalence of neurologic disease among infants with PVFM, most notably the 54% reported in the largest cohort study argues against a coincidental association and instead supports a causal link between neurologic dysfunction and paradoxical vocal fold motion.^15^

The neuroanatomic relationships found in various studies also support this mechanistic relationship. The affected lesions are invariably centered on the laryngeal motor control network which comprises of the brainstem respiratory centers, nucleus ambiguus, vagal efferent pathways, basal ganglia and supranuclear cortical areas. Several disorders of the brainstem, either structural (Chiari malformations and hydrocephalus) or functional (MSA), proved to be the most stable predictors of inspiratory vocal fold adduction.^35^^,^^36^ These results are consistent with known neurophysiology in which the abduction of inspiratory vocal folds is mediated by the posterior cricoarytenoid muscle which is operated by the vagal motor and regulated by the supranuclear and basal ganglia pathways.^37^^,^^38^ Interruption in this axis at any stage can cause adduction to be inappropriate during the inspiration, which causes the typical stridor and airway blockage as noted in PVFD.^39^

This review also gave a clear representation of the peripheral mechanisms. Cases of myasthenia gravis and paramyotonia congenita illustrate how neuromuscular junction dysfunction or ion-channel abnormalities can selectively impair laryngeal abductor function, leading to fatigable or episodic inspiratory obstruction.^24^^,^^29^ In these conditions, paradoxical vocal fold motion is best understood not as abnormal laryngeal behavior, but as a direct consequence of impaired neuromuscular transmission or altered muscle excitability.^40^ The preservation of normal expiratory abduction and phonation in many of these cases further supports a selective vulnerability of inspiratory control mechanisms, rather than global laryngeal paralysis.

On a broad comparison with the PVFD literature, there is evident dissimilarity between neurogenic PVFM, and psychogenic or functional PVFD. Earlier literature theorises that PVFM is a maladaptive or stress related laryngeal response, which is usually determined following the exclusion of structural pathology.^17^^,^^41^ These findings support a neurogenic subgroup of PVFM, particularly in infants and atypical cases. A systematic and neurologically oriented diagnostic approach is therefore essential in patients with suspected PVFM, particularly in those with atypical presentations, refractory symptoms, early onset, or associated neurological features.^42^ The cornerstone of diagnosis remains flexible fiberoptic laryngoscopy performed during symptomatic episodes, which directly demonstrates paradoxical inspiratory adduction of the vocal folds with preserved or near-normal expiratory abduction.^43^ This objective visualization is critical not only for confirming the diagnosis of PVFM, but also for distinguishing it from vocal fold paralysis, structural laryngeal lesions, subglottic stenosis, and lower airway disorders such as asthma.^17^ In many patients, especially those initially labeled as having difficult or steroid refractory asthma, laryngoscopic confirmation serves as the pivotal diagnostic step that redirects the clinical pathway toward appropriate neurological evaluation.^41^

Neuroimaging, particularly magnetic resonance imaging of the brain and brainstem, is of primary importance in patients with persistent stridor, feeding difficulties, apneic spells, cranial nerve deficits, or early onset disease.^44^ Structural abnormalities affecting the posterior fossa or brainstem, such as Chiari malformations, hydrocephalus, or space-occupying lesions, may directly compromise the nucleus ambiguus, vagal efferent pathways, or respiratory pattern generators, thereby producing paradoxical inspiratory adduction.^45^ Early identification of such lesions is clinically significant, as several conditions reported in this review demonstrated substantial or complete resolution of PVFM following neurosurgical decompression or cerebrospinal fluid diversion.

In patients with suspected peripheral or neuromuscular etiologies, electrophysiological studies may provide additional diagnostic clarity. Electromyography and nerve conduction studies can help identify disorders affecting neuromuscular transmission or muscle excitability, such as myasthenia gravis or ion-channel disorders.^46^ Recognition of these mechanisms is clinically important, as disease-specific therapies such as anticholinesterase agents or targeted pharmacologic treatment of channelopathies have been associated with normalization of vocal fold motion in reported cases.^32^ Similarly, in patients presenting with episodic respiratory distress, transient aphonia, or events suggestive of altered consciousness, electroencephalography should be considered to evaluate for seizure related PVFM.^4^ Several cases in the literature have demonstrated laryngeal dysfunction as a manifestation of focal epileptic activity, particularly in pediatric populations.^14^ In these scenarios, appropriate antiepileptic therapy may lead to resolution of paradoxical vocal fold motion, further supporting the causal relationship between neurologic dysfunction and laryngeal motor control abnormalities.^14^^,^^15^ Pulmonary function testing with flow volume loop analysis may serve as a supportive, though non-specific, diagnostic modality.^47^ During symptomatic periods, patients may demonstrate features of variable extrathoracic airway obstruction, characterized by flattening of the inspiratory limb of the flow volume loop. However, spirometric findings are often normal between episodes and cannot reliably distinguish neurogenic from functional PVFM.^48^ Consequently, pulmonary function testing should be regarded as an adjunct rather than a definitive diagnostic tool. Taken together, these findings support a multidisciplinary diagnostic strategy integrating laryngoscopic confirmation with targeted neurologic assessment. Collaboration among otolaryngologists, neurologists, pulmonologists, and, when appropriate, neurosurgeons is essential to ensure accurate diagnosis, identification of underlying neurologic pathology, and timely initiation of disease-specific management.^49^ Such an approach not only reduces misdiagnosis and inappropriate respiratory therapies, but also enables early detection of potentially reversible neurologic conditions, thereby improving both airway and neurological outcomes.

The results of treatment across included studies that further support the relationship between neurologic pathology and PVFM. Table 3 presents a summary of all reversible or curable neurologic conditions (epilepsy, hydrocephalus, Chiari malformation, myasthenia gravis and sodium-channel disorders) where PVFM significantly improved or vanished with specific intervention. Decomposition of the posterior fossa and cerebral spinal fluid diversion, especially in children with Chiari malformations, were found to be particularly effective and would result in the long-term normalization of airway function and prevent tracheostomy.^16^^,^^23^ Likewise, PVFM like episodes were resolved in cases of seizures using antiepileptic therapy, and PVFM was restored to normal motor vital fold movement in myasthenic and channelopathy-related PVFM cases.^14^ In contrast, outcomes differed substantially in progressive neurodegenerative conditions. In MSA, continuous positive airway pressure improved stridor and nocturnal airway patency but did not eliminate paradoxical vocal fold motion, reflecting irreversible degeneration of brainstem and basal ganglia laryngeal control circuits.^25^^,^^27^ This distinction between reversible and progressive neurological disease has important prognostic implications and reinforces the concept of PVFM as a marker of underlying neurologic disease severity and trajectory.^50^ Supportive airway interventions, including CPAP and botulinum toxin injections, were valuable adjuncts in selected cases but did not substitute for disease-specific neurologic management.^24^, ^25^, ^26^, ^27^^,^^30^

The implications of these findings on clinical practice are significant. Lack of recognition of neurogenic PVFM may result in recurrent misdiagnosis asthma, inappropriate escalation of corticosteroid or bronchodilator therapy, and unnecessary invasive airway interventions.^51^^,^^52^ Recognizing PVFM as a possible neurological manifestation rather than a diagnosis of exclusion may facilitate earlier neurologic evaluation, appropriate imaging, and timely multidisciplinary management. Despite these strengths, the evidence base synthesized in this review has important limitations. The predominance of case reports and small observational studies introduces inherent risks of selection bias, publication bias, and heterogeneity in diagnostic and outcome measures. The rarity of neurogenic PVFM and variability in laryngoscopic techniques, neurologic evaluations, and follow up durations precluded quantitative meta-analysis. However, given the mechanistic coherence of findings across diverse conditions and the consistent temporal relationship between neurologic intervention and PVFM outcomes, qualitative synthesis was both appropriate and informative. Importantly, risk-of-bias assessments were used to contextualize findings rather than exclude studies, preserving valuable data from rare but clinically instructive cases. This review has several notable strengths. To our knowledge, it represents the first systematic synthesis focused specifically on organic neurological causes of PVFM, integrating neuroanatomic correlations, clinical phenotypes, and treatment outcomes across age groups. By adopting a neurologically informed framework, this review bridges gaps between neurology, otolaryngology, and respiratory medicine, and provides a foundation for improved diagnostic reasoning and interdisciplinary care.

Future research should prioritize prospective studies with standardized diagnostic criteria, systematic neurologic assessment of PVFM patients, and longitudinal outcome reporting. Advanced neuroimaging and neurophysiologic techniques may further elucidate the precise neural circuits involved in laryngeal motor control. Establishing PVFM as a recognized neurologic manifestation rather than a purely functional diagnosis may ultimately improve patient outcomes and reduce healthcare burden.

The principal contribution of this review is not to establish the prevalence of neurological disease among all patients with PVFM, but to define a clinically meaningful neurogenic subgroup. By integrating neuroanatomic localization, clinical presentation, and treatment response, this review provides a framework for recognizing when PVFM should prompt neurological evaluation and when disease-specific treatment may alter airway outcomes.

### Recommendations

**Fig. 4:**
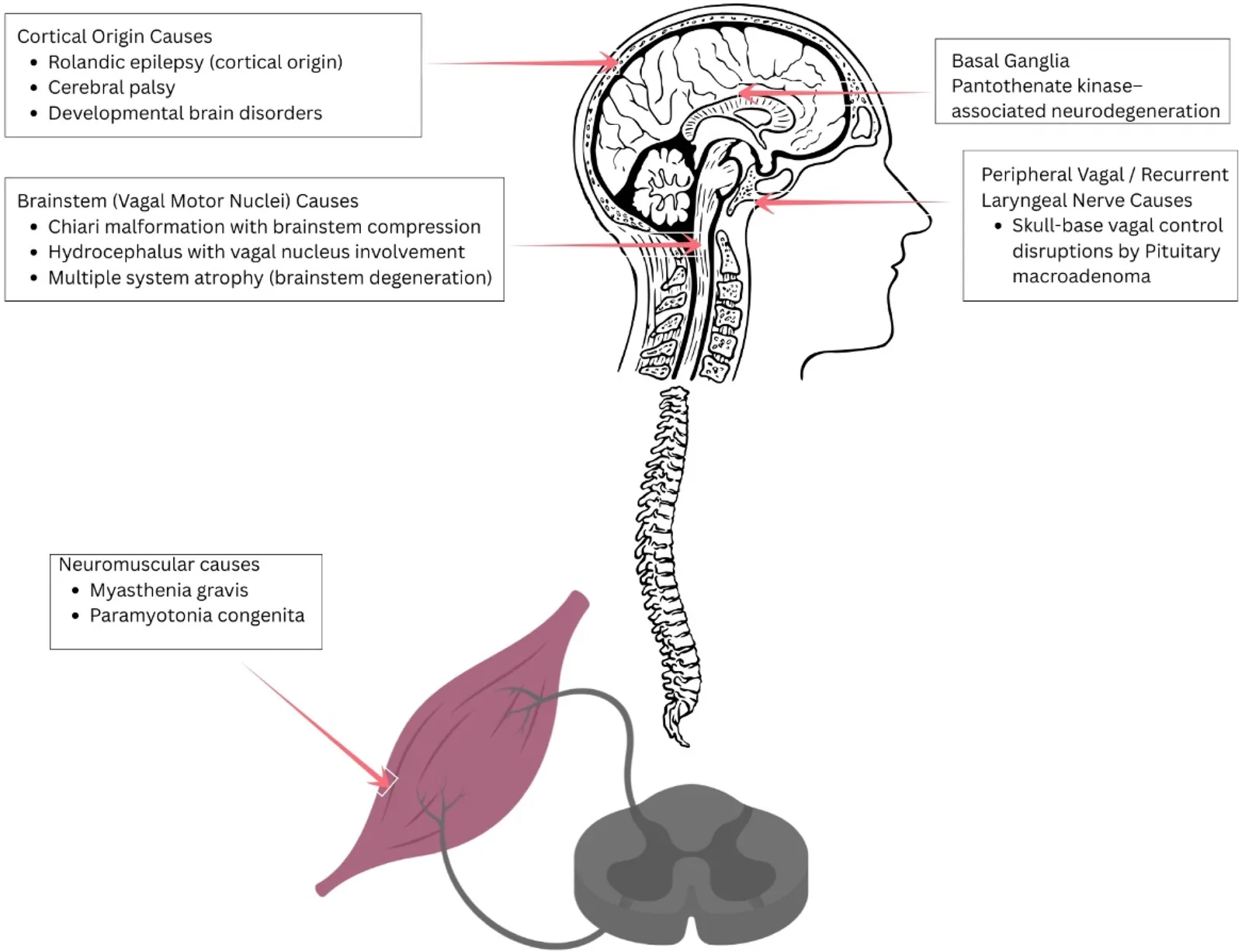
Neuroanatomical localization of neurological causes of paradoxical vocal fold motion.

Based on the consistent association between paradoxical vocal fold motion and underlying neurological disorders observed across the included studies, a structured and neurologically oriented diagnostic approach is recommended. PVFM should not be regarded solely as a functional or psychogenic condition, particularly in patients with atypical presentations, early onset, refractory symptoms, or associated neurological signs. Instead, clinicians should consider PVFM as a potential manifestation of disrupted neural control of the larynx across multiple levels of the nervous system. The neuroanatomical substrates implicated in neurogenic PVFM including cortical, basal ganglia, brainstem, peripheral vagal, and neuromuscular pathways ‒ are summarized in Fig. 4, highlighting the distributed neural network responsible for laryngeal motor control.

**Fig. 5:**
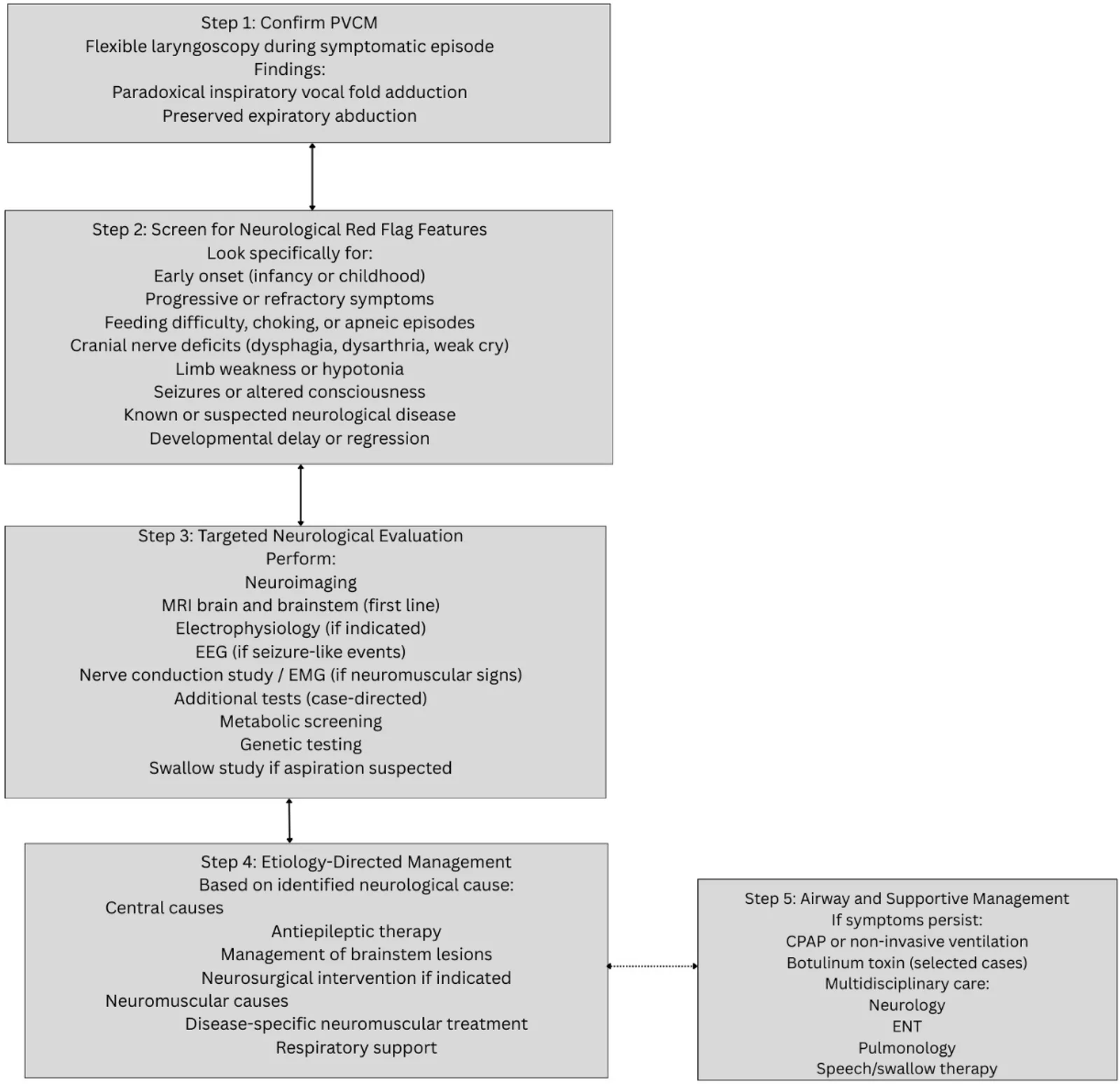
Algorithm for the management of neurological paradoxical vocal cord motion or dysfunction.

To translate these mechanistic insights into clinical practice, a stepwise diagnostic and management algorithm is proposed in Fig. 5. This algorithm emphasizes early laryngoscopic confirmation, systematic screening for neurological red-flag features, targeted neuroimaging and electrophysiological evaluation when indicated, and etiology directed treatment alongside supportive airway management. Adoption of such a structured approach may reduce misdiagnosis, facilitate early detection of reversible neurological conditions, and improve both respiratory and neurological outcomes.

## Conclusion

PVFM can occur in association with organic central and peripheral neurological disorders, particularly involving brainstem, vagal, basal ganglia, supranuclear, neuromuscular junction, and ion-channel pathways. Although the available evidence is limited mainly to case reports, case series, and small observational cohorts, improvement after treatment of reversible neurological conditions supports a clinically relevant neurogenic subgroup. Patients with early-onset, atypical, refractory, or neurologically associated PVFM should undergo targeted neurological evaluation, while management should combine standard PVFM therapy with disease-specific treatment when an underlying neurological disorder is identified.

## ORCID ID

Rashmi Gupta: 0009-0005-2526-6002

## Authors’ contributions

Bigyan Raj Gyawali: Conceptualization; data curation; formal analysis; methodology; project administration; original writing, review, editing, and visualization.

Bibek Shrestha: Conceptualization; data curation; formal analysis; methodology; project administration; original writing, review, editing, and visualization.

Shreeram Paudel: Resources; validation; conceptualization; investigations, and data curation.

Rashmi Gupta: Resources; validation; conceptualization; investigations, and data curation.

## Consent for publication

Not applicable.

## Ethics approval and consent to participate

Not applicable. This study is a systematic review of previously published literature and did not involve direct participation of human subjects or collection of new patient data.

## Funding

No specific funding was received for this study.

## Data availability statement

The authors declare that all data are available in repository.

## Availability of data and materials

All data generated or analyzed during this study are included in this published article and its Supplementary Materials.

## Declaration of competing interest

The authors declare no conflicts of interest.
